# The Estradiol-Dihydrotestosterone model of prostate cancer

**DOI:** 10.1186/1742-4682-2-10

**Published:** 2005-03-18

**Authors:** A Edward Friedman

**Affiliations:** 1 Department of Mathematics, University of Chicago, 5734 S. University Avenue, Chicago, IL 60637, USA

## Abstract

**Background:**

The exact relationship between hormonal activity and prostate cancer(PCa) has not yet been clearly defined. One of the key hormones associated with PCa is testosterone(T). However, both *in vitro *and *in vivo *studies have shown that under some conditions T is capable of either promoting PCa growth or death. This article proposes a theory which resolves this apparent paradox.

**Model:**

The Estradiol-Dihydrotestosterone(E-D) model introduced in this paper proposes that 17β-estradiol(E2) is essential for initiating the growth of PCa cells through the formation of telomeres. It also proposes that T is responsible for increasing the expression of proteins which cause apoptosis, or programmed cell death, and that 5α-dihydrotestosterone(DHT) is essential for preventing this. In addition, it is known that some T is converted to both E2 and DHT, which means that depending on the conditions, T is capable of either promoting the growth of or the killing of PCa.

## Background

There are currently two models for prostate cancer(PCa) which are diametrically opposed to each other. One model[1] proposes that high levels of androgens are responsible for PCa. This model is unable to explain the fact that androgen levels drop with age while the incidence of PCa increases. The other model[2] proposes that high levels of androgen should be effective in the prevention and treatment of PCa. While this model is consistent with the relationship of PCa to age, it does not explain experiments in which increased levels of androgen have been shown to increase the incidence of PCa, such as the correlation of increased incidence of PCa with higher levels of free testosterone(T)[3].

## Model

### Model description

The Estradiol-Dihydrotestosterone(E-D) model of PCa, proposed in this paper, hypothesizes that 17β-estradiol(E2) is essential for turning on telomerase in PCa, resulting in telomere formation. It is hypothesized that it does this by binding to a heterodimer composed of one estrogen receptor-α(ER-α) and one estrogen receptor-β (ER-β). This model proposes that telomere formation is an essential step for initial PCa growth. It also hypothesizes that 5α-dihydrotestosterone (DHT) is essential for initial PCa growth by binding to the intracellular androgen receptor(iAR), which results in the cell being protected from the apoptosis caused by androgens binding to the membrane xandrogen receptor(mAR). Finally, it proposes that T binds to mAR and upregulates proteins that promote apoptosis, such as U19, ALP1, and Fas.

All of the above hormones also have other interactions which affect PCa. The E-D model proposes that when T binds to mAR, it upregulates bcl-2, a protein that protects against apoptosis, whereas when DHT binds to iAR, bcl-2 is downregulated. It is hypothesized that the expression of bcl-2 increases when E2 binds to ER-α and decreases when E2 binds to ER-β. It is known that T is converted to E2 by the enzyme aromatase(Aro) and is converted to DHT by the enzyme 5-α reductase type II(5AR2).

### Supporting evidence

It has been observed that mice never get PCa if they have a genetic mutation resulting in a lack of ER-α[4] or Aro[5]. These findings are consistent with the E-D model, which would also predict that mice with a genetic mutation resulting in a lack of ER-β would never get PCa.

Aro activity was not observed[5] in normal epithelial prostate cells, but was observed in all of the PCa cell lines tested. E2 has been shown[6] to turn on telomerase activity, with both ER-α and ER-β being involved, in human epithelial cells as well as in tissue cultures of PCa. Some researchers believe that telomerase activity is an essential step for all types of cancer[7]. These findings are consistent with the prediction of the E-D model that E2 is essential for the initiation of PCa growth by bringing about telomere formation.

Implantation of PCa xenografts into intact and ovariectomized female mice showed that E2 inhibited the growth of 5 out of the 6 xenografts tested[8]. The authors concluded that "the observed inhibition of PCa growth may be attributable to direct effects of estrogens via ER-β". Mice with a genetic mutation lacking ER-β[9] have an overexpression of bcl-2 in their ventral prostate. E2 has been shown[10] to increase the production of bcl-2 in MCF-7, an ER-α positive cell line of breast cancer. This increase is negated by the addition of 4-hydroxytamoxifen(OHT). It is known[11] that OHT acts as an antagonist to ER-α. All of this is consistent with the hypotheses of the E-D model that E2 increases the production of bcl-2 when binding to ER-α and decreases it when binding to ER-β.

It has been shown[12] that T binds to mAR to produce apoptosis by upregulating the protein Fas. In the PCa cell line DU145 which lacks iAR, T or DHT alone was sufficient to induce apoptosis. However, in LNCaP, a hormone sensitive PCa cell line which has a functional iAR, T promotes cell growth unless it was given in the form of T-BSA, which does not enter the cell nor bind to the iAR, in which case apoptosis was also induced. This shows that the binding of androgens to iAR can counteract the apoptosis otherwise induced by the binding of androgens to mAR, as proposed in the E-D model.

It has been shown[13] that T upregulates a protein, U19, which induces apoptosis in PCa. This apoptosis was inhibited by mibolerone, a synthetic androgen, binding to the iAR. Another protein, ALP1, that was upregulated by T, was also found[14] to induce apoptosis. These findings are all consistent with the E-D model.

When finasteride(F), an inhibitor of 5AR2, is added to LNCaP, it inhibits growth in a dose-dependent manner[15]. Apoptosis resulted at the highest dose tested. This indicates that T binding to iAR is not able to completely prevent the apoptosis induced by T binding to mAR, whereas DHT is. This can be represented symbolically as T:mAR >>> T:iAR in the presence of F and DHT:iAR >>> T:mAR in the absence of F. This is consistent with the hypothesis of the E-D model that DHT is essential for initial PCa growth because it protects the PCa from mAR induced apoptosis.

It is known[1] that men with a genetic mutation that produces non-functional 5AR2 do not get PCa. Since 5AR2 is found within the prostate cells and converts T to DHT, the result is very little DHT in the prostate. This finding is consistent with the hypothesis that in the absence of DHT, T binding to iAR in PCa is not able to prevent the apoptosis caused by T binding to mAR.

It has been shown[16,17] that in a small percentage of men with castrate metastatic PCa, enormous improvement in symptoms occurred following the administration of T. One possible explanation for this is that the PCa in those individuals might have lacked a functional iAR, but retained a functional mAR. The fact that T alone is sometimes capable of causing apoptosis *in vivo *is consistent with the E-D model.

When pyrrolidinedithiocarbamate(PDTC), a strong anti-oxidant, is added to LNCaP, it induces apoptosis[18]. It was shown that when 10^-12 ^M T was added to LNCaP, it increased the growth rate, but when it was added in addition to PDTC, the amount of apoptosis was greater than that induced by PDTC alone. This again shows that T is capable of inducing apoptosis under the proper circumstances.

T has been shown[12] to increase bcl-2 production when it binds in the form of T-BSA to the mAR of LNCaP. T-BSA was shown not to bind to iAR. DHT has been shown[19] to decrease bcl-2 production when it binds to the iAR of LNCaP-FGC. This decrease was inhibited by the addition of bicalutamide, an antiandrogen which interferes with the binding of DHT to iAR. This is consistent with mAR being involved in the upregulation of bcl-2 and iAR being involved with the down-regulation of bcl-2, as proposed in the E-D model.

## Discussion

The E-D model, presented in this paper, does not explain how genetic mutations occur that cause PCa, but does explain which factors are essential for PCa to grow. The crucial factors for initial PCa growth are telomere formation and apoptosis avoidance. Since E2 produces telomere formation[6], in the absence of high levels of exogenous E2, Aro activity would initially be needed to supply PCa with large amounts of E2. The binding of normal amounts of DHT to iAR is sufficient for PCa to initially avoid apoptosis. As mutations develop that interfere with the process of apoptosis, DHT binding to iAR may no longer be necessary.

The evidence is overwhelming that T is capable of inducing apoptosis in PCa. The *in vitro *and *in vivo *studies are unambiguous in this regard. The study with PDTC[18] raises interesting questions that can only be answered with further experimentation. There are three possible explanations as to why T increased the apoptosis caused by PDTC. Either PDTC interfered with the binding of androgens to iAR, or PDTC inhibited 5AR2, or PDTC may have enhanced the ability of T to cause apoptosis when binding to mAR. If the latter is true, it would have important implications in preventing and treating PCa, especially if other anti-oxidants should also be found to exhibit this same sort of enhancement.

Assuming that the genetic studies with mice are applicable to humans, then it is clear that E2 is essential in order for PCa to occur. The evidence is only circumstantial that E2 is binding to a heterodimer of ER-α and ER-β. If a dimer were not involved, then one would expect that small amounts of E2 would be sufficient to induce telomerase activity. The heterodimer is consistent with the observation that both ER-α and ER-β are involved in telomere formation[6]. The hypothesis that telomere formation is essential for all cancers to occur[7] is not a proven fact, but it gives a plausible explanation as to why E2 is initially essential for PCa.

The genetic study in mice[9] makes it clear that ER-β acts to inhibit bcl-2 production. The evidence that ER-α increases bcl-2 production is more circumstantial. It is known[20] that ER-α and ER-β tend to counteract each other. The increase in bcl-2 in the breast cancer line exposed to E2[10] is consistent with ER-α being responsible for increasing bcl-2. However, this assumes that the effect remains the same when the same hormone binds to the same hormone receptors in breast and prostate cancers. This is an intriguing concept, and would imply that the different overall effects that hormones have on these two cancers could be explained by the different amounts of each hormone receptor present in them. More research is needed to learn if breast cancer also possesses mAR and iAR acting in opposition to each other, as they do in PCa.

High dosages of E2 have been used in the treatment of PCa. Transdermal patches of E2 have been shown to produce castrate levels of T within 3 weeks[21]. It is known that castrate levels of T result in apoptosis of most PCa cells, with calcium overload being one of the causes[22]. Calreticulin protects prostate cells from apoptosis due to calcium overload by enhancing the calcium buffering capacity, but castrate levels of T dramatically downregulate calreticulin. More research is needed to determine whether calreticulin is upregulated by mAR, iAR, or both.

The fact that Aro activity was found in PCa cell lines, but not in normal prostate cell lines[5], does not mean that it is present in all PCa. Even if E2 is necessary for telomere formation, once telomeres of sufficient length are formed, Aro activity may no longer be necessary. It is interesting to note that the level of Aro activity observed in the PCa cell lines falls within the range observed in breast cancer cell lines[5].

The assumption of the E-D model that when DHT binds to iAR it counters the effect of apoptosis induced when T binds to mAR is supported by the study[15] that showed that increases in F decreased the growth of LNCaP in a dose-dependent manner, with the highest dose causing apoptosis. If the behaviour *in vivo *is the same as this *in vitro *result, then it explains why men with defective 5AR2 would not get PCa[1].

There are many questions still to be answered about PCa. Do the progesterone(P) receptor isomers, PRA and PRB, affect bcl-2 production? What is the dose effect of T, DHT, E2, and P on each of their corresponding receptors? For the hormone receptor pairs, to what extent does each receptor work against the other?

Another important issue is how mAR is involved in the apoptosis of aged normal prostate cells. The fact[12] that T is capable of causing apoptosis in PCa which lacks iAR indicates that the increased amount of bcl-2 produced by mAR is not sufficient to counteract the apoptotic proteins produced by mAR. The makes it likely that the production of bcl-2 by mAR represents a damping factor designed to protect normal prostate cells from inappropriate apoptosis that might otherwise be caused by local fluctuations in serum T levels. If it turns out that calreticulin[22] is upregulated by mAR, then the absence of mAR should lead to apoptosis through calcium overload. As normal prostate cells age, if they start to lose functionality of mAR, iAR, or both, then the probability of apoptosis occurring should increase as the amount of functional AR decreases.

It is beyond the scope of this paper to develop detailed descriptions of treatments for preventing or treating PCa. However, it is clear that treatments that maximize the pro-apoptotic properties of mAR, minimize the anti-apoptotic properties of iAR, and minimize overall bcl-2 production should be of therapeutic value. An example of this would be a combination of T, F, and methyl-piperidino-pyrazole(MPP) along with avoiding foods known to have components that selectively bind to ER-β. MPP has been shown[23] to be an antagonist of ER-α but not of ER-β.

## Conclusion

The E-D model presented here is consistent with known experimental data. Further research is needed to more completely verify and expand its hypotheses. It explains how T can promote either PCa growth or apoptosis and why E2 and DHT are essential for initial PCa growth.
